# The Tropics on Our Doorstep
*A paper given by invitation to the Bristol Medico-Chirurgical Society on 10th February, 1954.


**Published:** 1954-07

**Authors:** George R. McRobert


### THE TROPICS ON OUR DOORSTEP*

BY

SIR GEORGE R. McROBERT, *C.I.E.*, M.D., F.R.C.P.

At the first international congress on medical education held in London in August 1953 the Royal Society of Tropical Medicine and Hygiene presented a memorandum on the need for instruction in tropical medicine to students intending to practise in temperate climates. When the subject was announced the majority of the audience left the room " for a breather ". What, thought they, could tropical medicine possibly mean to men whose work lay in Northern Europe?

### THE RISKS TODAY

Since the New Year the Mark II Comet has flown from London to Khartoum in six hours. It is now possible to leave London in the early morning, to touch down in tropical Africa, to spend some time bathing in delectable limpid freshwater pools, to disport oneself on a sandy beach, to rest under shady trees, to enjoy green salads and raw fruits at a picnic lunch and to return home the same night incubating, among many other infections, the malarias, the dysenteries, typhoid and typhus fevers, relapsing fever, hookworm disease, bilharzial infections of the bladder and bowel, sleeping sickness, loaiasis, onchocerciasis, subcutaneous maggots, and jigger fleas in the soles of the feet—a truly formidable problem in diagnosis to confront a general practitioner in Great Britain where there is no insect-borne disease amongst humans, virtually no protozoal infections, and with worm infestations practically confined to the lumen of the alimentary canal.

The practitioner in this country is increasingly liable to meet with hitherto unexpected problems in diagnosis, for apart from the theoretical much-afflicted tropical tripper to whom I have just referred thousands of visitors come by air each year from India, Africa and South America, all potentially incubating tropical maladies which, in the past, generally became manifest on the sea voyage to England.

The title of my talk is, I think, an apt one. The tropics are virtually on our doorstep or, at most, next door. The aftermath of the recent war, too, has brought to this country enormous numbers of men and women who contracted one or more tropical diseases in Burma, Africa or the Middle East, and as the correspondence columns of our journals indicate, the recent operations in Korea have let loose on our doctors here, numbers of young National Servicemen who contracted benign tertian malaria in Korea and who produced the first symptoms of it many months after their arrival home. We have, further, a great influx of Africans and West Indians to swell the labouring population of this Welfare State, and every part of our tropical Commonwealth sends a quota of students to universities, inns of court, technical institutes and schools, so raising still higher the amount of tropical disease to be diagnosed in this country.

It is, of course, impossible for me to deal adequately with this enormous subject in the time at my disposal. I propose to deal only with the malarias, amoebiasis, bilharziasis, loaiasis, onchocerciasis and leprosy. You may have noted that in my grim

*A paper given by invitation to the Bristol Medico-Chirurgical Society on 10th February, 1954.

catalogue of possible infections I did not mention smallpox and yellow fever. Against these two virus infections we can give absolute protection. Great care is taken by all airlines in checking in detail the vaccination and inoculation history of each passenger, and these two diseases give rise to little anxiety. The development of a perfect attenuated vaccine of the yellow-fever virus, causing no reaction of any kind, no painful swelling on injection, and giving complete protection within ten days of administration and for six years thereafter against the dreaded yellow jack which used to terrorize all mariners sailing from this and other ports to West Africa and the Spanish Main encourages us to hope that before long the viruses of poliomyelitis and encephalitis will be successfully mastered. I find the daily procession of peoples of all nations, colours, creeds and ages (seven days old to ninety years) attending our daily yellow-fever inoculation clinic most interesting, stimulating and thought provoking.

### MALARIA

Malaria in human beings is caused by four species of unicellular animals or protozoa of the genus *Plasmodium*, but from the clinician's point of view there are two types of malaria:

The vital-organ sporulators—malignant malaria;

The surface-capillary sporulators—the benign malarias.

The essential difference between the two types is that in malignant or falciparum malaria the multiplication or sporulation of the parasites within the red blood cells takes place within the capillaries of the vital organs in the depths of the body giving rise to multiple blocking of capillaries with acute infarctive and anoxic phenomena in the brain, pituitary and suprarenals, bowel, heart or kidneys resulting according to the organ mainly affected in deep coma, choleraic diarrhoea, precordial pain and collapse or massive shock. Death may be almost immediate.

In the benign malarias the sporulation takes place in the surface capillaries so that although the benign tertian, ovale and quartan forms of malaria are debilitating and exhausting, they are not acute emergencies and are not immediately dangerous.

### RECENT DISCOVERY ABOUT LIFE-CYCLE

I should like at this point to spend a few moments dealing with the life-cycle of the malaria parasites. If you refer to the text-books on your shelves you will find in all but the most recent editions an account of how the female anopheline mosquito injects malaria parasites with her salivary fluid as from a hypodermic syringe and that these minute torpedo-shaped sporozoites enter the red blood cells and multiply: when a sufficient number of red cells are invaded and ruptured symptoms become manifest with rigors, flushes and fever. Fifty years ago Schauddin said that he saw the sporozoites enter red blood cells in a wet preparation under a microscope. He was completely mistaken. The world was misled for half a century. The parasites injected by the mosquito do not enter the corpuscles at all. During the recent war my present colleague Sir Neil Hamilton Fairley working on human volunteers in Australia showed that the blood of Smith and Brown who had been bitten by heavily infected mosquitoes, if withdrawn and subinoculated into Jones and Black would produce malaria in them only if the period between the mosquito biting and the withdrawal into the syringe was less than thirty minutes or more than six days. In other words the sporozoites are present in the circulatory blood for a brief period after the mosquito bite—in the plasma—but must rapidly disappear to some unknown destination from which infective forms emerge and are seen in red blood cells about a week later. Where they had gone no one knew till Henry Shortt and his co-workers discovered the liver cycle of malaria parasites in 1948/49/50, first in monkeys and later in human volunteers in benign tertian and malignant malaria. We now know that the malaria parasites enter and multiply in the polygonal cells of the liver before invading the red blood cells and that what we now call Shortt's tricycle has three parts:

(1) In the mosquito;

(2) In the liver;

(3) In the red blood cell.

From the points of view of prevention and treatment a very important difference has been discovered between malignant falciparum malaria and the benign malarias, and it is greatly to Hamilton Fairley's credit that he predicted this difference on a theoretical basis before Shortt demonstrated it by experiment. The parasite of malignant malaria visits the liver and leaves completely after a few days. The parasites of benign malarias lodge in the liver and merely send out periodic detachments into the blood.

*Malignant malaria* has been the No. 1 Killer in the tropics in the past and is still the most important disease in the world and is the chief problem facing hygienists and medical administrators in the tropics. So far as visitors to the tropics are concerned, soldiers, sailors, airmen, commercial travellers and tourists—and in fact in all organized labour forces—the problem may be said to be a vanishing one.

*Treatment.* Thanks to modern synthetic drugs we have absolute power to prevent the development of malignant malaria as certain synthetics, taken in small doses daily, kill the liver-forms before they can develop to the blood-invading stage and are what is known as causal prophylactics.

Paludrine (Proguanil) is the outstanding example. No European going to the tropics should contract malignant malaria. Blackwater fever *should* be a thing of the past—and very nearly is. We should never see malignant malaria in this country if all persons passing through malarious areas were to take their daily prophylactic pill during the period of risk and for a fortnight thereafter. But tragedies continue to occur—chiefly amongst the occasional or casual traveller passing through Africa from one temperate zone to another, from Britain to Cape Town for example. A mosquito bite in Nigeria may cause an acute seizure, hyperpyrexia, coma and collapse in Bristol or Johannesburg, and unless the practitioner is ready with his syringe all may be over in a very short time.

When acute symptoms develop in one recently returned from the tropics—take a blood slide (a thin and thick film and a thick drop if possible). Have it examined by an expert at leisure but do give a potent antimalarial drug as soon as possible by mouth if the patient can swallow readily—intramuscularly or intravenously if he cannot.

Those of you who have served in the forces are probably familiar with the German antimalaria weapon Atebrin (Mepacrin) which enabled us to defeat the Japanese in South-East Asia. It is still a valuable drug, but its German successor Chloroquin (Nivaquin, Resochin, Aralen) is probably the best drug for the treatment of malaria.

The prophylactic drugs Paludrine (Proguanil) and Daraprim have no place in the treatment of acute malaria in Europeans. They come into action much too slowly.

Please take a blood slide if you can, but don't wait for the result before starting to treat the acute disease.

*Benign malaria.* I now pass to the benign oft-relapsing malarias of which we are seeing a great deal in Britain today.

A fortnight after leaving the tropics the visitor usually stops taking his suppressive paludrine or daraprim—malignant malaria is ruled out as a future possibility but the liver may contain dormant or smouldering forms of the benign malarias. Many months later an attack of malaria may occur even if no clinical malaria had occurred in the tropics. That is the usual story in the young servicemen who are going down with malaria in Britain today. Some of our patients have been treated for weeks or months with antibiotics, sulphonamides and diaphoretic mixtures, arriving in hospital greatly debilitated and with large spleens. Paludrine or daraprim kept the malaria suppressed, and whilst taking one tablet a day no clinical symptoms will occur.

### TREATMENT OF RELAPSES OF BENIGN MALARIA

We now know certain drugs of the 8-amino-quinoline series which do act on the liver forms of the benign malarias. The earlier numbers of the series — the German plasmoquin (pamaquin) were rather toxic in even small doses, but the latest American production Primaquin is notably less toxic and more efficient. Given in small doses for a fortnight it destroys the liver-forms of benign malaria in a high proportion of cases. The American Army has been giving this drug to returning troops on the voyage across the Pacific to the U.S.A., and the incidence of benign tertian malaria is now very low in Korean veterans in the U.S.A. and Canada. Primaquin has recently become available on this side of the Atlantic. It is not suitable for use in general practice—the patient should be under close supervision during the whole course of administration.

### AMOEBIASIS

I now pass on to consider briefly the subject of infectic ns with *Entamoeba histolytica* which give much trouble to practitioners in this country. We have more references from general practitioners for consultation at the Hospital for Tropical Diseases for chronic bowel disease than for any other complaint: the history given by the patient is generally that the trouble started on a trip to South Italy, Spain or Greece or, of course, on war service in the Middle or Far East. The subject is not any easy one to deal with—even the parasite itself is not fully understood.

The cause of amoebic dysentery is a freely motile grey protozoon—*Entamoeba histolytica*—with well-defined ectoplasm, rapidly protruded pseudopodia, apparently purposive movement over the microscope field and containing in its protoplasm several red blood cells in process of digestion. It burrows into the submucous coat of the colon —chiefly in the caecum, but in heavy infections extending right down to the lower rectum. In the submucous layer by a process of lysis and gelatinous degeneration the characteristic flask-shaped ulcers are formed with narrow ulcerated openings to the surface. Between the ulcers the lining of the colon is surprisingly normal in appearance. The amoebae enter radicles of the portal vein, travel to the liver and may reach the lungs, brain, muscles and other parts of the body causing far-reaching effects.

*Entamoeba histolytica* can be cultured quite easily on artificial media but a bacterium —such as *B. coli*—must be grown with it. It cannot be grown alone.

Much speculation at present rages with regard to the pathogenicity of *E. histolytica* by itself. Does it require a second organism to enable it to penetrate the gut wall? Do secondary infections determine the severity of the intestinal lesions? It has been generally taught that amoebic dysentery has a mild onset in contra-distinction to the acute start of bacillary dysentery, but one meets cases which start in a fulminating fashion, with high fever, severe constitutional symptoms and sloughing of the gut wall. Many now believe that it is secondary invaders which are responsible for such a picture.

The characteristic four-nucleate cysts of *E. histolytica* may be found in the stools of inhabitants of these islands who have never left these shores—recently an investigation at a school for mental defectives revealed an astonishing percentage of infections. It may be said, however, that the cysts passed by such non-travellers are under $10\mu$ in diameter, whereas cysts in the tropics generally slightly exceed that measurement. It is probable that there exists here and elsewhere a harmless smaller form of *E. histolytica* living in the bowel contents as a harmless commensal and with no invasive tendencies.

*Management.* What is the practitioner to do if cysts of *E. histolytica* are reported in the stools of a person who has never been abroad? If there are no bowel symptoms and if the patient is not a food handler treatment is unnecessary. If the patient has been abroad one assumes that one is dealing with a pathogenic variety and full treatment at the hands of an expert is indicated.

*Drug treatment.* The sheet-anchor in the treatment of amoebiasis is emetine and its derivatives. The toxic nature of emetine has been over-emphasized but it is wise to keep the patient in bed whilst administering it. The place of the antibiotics Aureomycin, Terramycin and Fumigillin in the treatment of amoebiasis is not yet fully determined. It is probable that a combination of emetine with fumigillin is the best treatment for amoebic dysentery. It is quite clear that the use of antibiotics alone which, under the influence of high-pressure American salesmanship, have displaced emetine in better-class practice in certain parts of the tropics, is producing a crop of liver and lung abscesses, staphylococcal enteritis and mould infections of the gastro-intestinal tract which we did not see when we relied on emetine.

The invasion of the liver by many amoebae causes acute hepatitis which may proceed to the formation of one or more sterile liver abscesses—the largest of which is usually found in the right lobe of the liver. Since Sir Leonard Rogers introduced emetine therapy in 1910 it has been possible to cause amoebic hepatitis or even amoebic abscesses to resolve without recourse to surgery. If such intervention is necessary simple aspiration is generally sufficient. Open operation—so fatal in the tropics in pre-antibiotic days—is practically never necessary except in left-lobe abscesses pointing in the epigastrium or left hypochondrium. During the past year I have seen six patients who have been subjected to unnecessary open operation for liver abscesses treated by antibiotics without emetine.

*Chloroquin.* Only very occasionally is *E. histolytica* emetine-resistant, and we now have a very powerful second string in Chloroquin which I have already mentioned as the best synthetic drug for treating acute malaria. Chloroquin is highly concentrated in the liver and lung and deals efficiently with amoebae deposited there. It does not concentrate in the intestinal wall and so is useless for the treatment of the bowel infection.

Amoebae may produce lesions in the lung, brain, and skin, and granulomatous tumours of the bowel may be amoebic and may resolve readily when emetine is given. Lungs have been removed for amoebic abscess thought to be malignant. In a recent number of the *B.M.J.* a surgeon near London reported that he had operated on a tumour in the right iliac fossa suspecting malignancy and with most unfortunate results. The tumour was an amoeboma and the diagnosis was not suspected till amoebae invaded the skin from the colostomy. Let me say that in persons who have returned from the tropics a diagnosis of appendicitis, carcinoma of the colon or rectum should be arrived at only after amoebic infection has been excluded.

### SCHISTOSOMIASIS

We are very fortunate that in this country human beings have little to fear from snail-borne disease—an occasional accidental infection with the sheep fluke—*Fasciola hepatica*—and transient skin rashes in bathers in some freshwater reservoirs but nothing more.

Everyone knows that the chief curse of Egypt is bilharzial infection with resulting haematuria, contraction and often carcinoma of the bladder, gross splenomegaly, cirrhosis of the liver and ascites, but it is not so well known that both bladder and bowel varieties produce havoc in Egypt and the Soudan—the male and female worms living in the portal vein, their sharp-spined ova finding their way not only to their natural exits in the bladder and bowel but also wandering in the blood currents to sites in the lung, heart, brain and spinal cord in each of which pathological changes can be set up.

From your zoology days you may remember that the sharp eggs rupture in fresh water, that the motile parasite enters an appropriate snail, invades its liver where it undergoes a complicated series of changes. Infective forms—the free-swimming cercariae—penetrate the skin of a human being exposed to infected water, make their way to the portal veins where they grow, couple and produce eggs.

In Arabia and in Africa, from Egypt in the north to Natal in the south, millions of people are afflicted with schistosomiasis. We must not think of it as a disease of the poor who work knee deep in rice fields and swamps. It is a very real danger to visiting

Europeans. Anything which brings infected water into contact with the skin is potentially dangerous, e.g. an angler's wet fishing line, the brief contact with water whilst picking a fish or a shot duck out of water and, of course, untreated water used for washing or drinking. During the past year I have treated doctors and their wives—and picnic guests of those same doctors—who so far transgressed the rules of tropical hygiene as to indulge in freshwater bathing in an area of Nigeria where freshwater snails abound. At the present time I am treating a very heavy *haematobium* infection in a European who fell out of his canoe whilst duck-shooting and also three school-children who contracted the disease whilst on a flying visit to parents in Africa. Very many parents in the tropics now have their children out during school holidays. Malaria prophylaxis seems to be well carried out but the real and ever-present danger of schistosome infection is not fully grasped. School doctors and general practitioners need to be on the look-out for cases of mild dysuria and slight chronic diarrhoea. Ova may be found in the urine or faeces. Complement fixation tests or skin sensitivity reactions and the presence of an eosinophilia will help in the diagnosis.

*The treatment* of these infections has to be very thorough. Although much research has been done in the hope of producing satisfactory drugs for oral administration, those on the market do not produce the high rate of cure claimed by the makers. We still rely on intravenous sodium antimony tartrate—originally introduced by Christopherson early in the 'twenties. The treatment is tedious—especially in *S. Mansoni* infections—but the eventual results are good in cases taken early.

### IMPORTANT NEMATODES

Now I want briefly to deal with three important nematode worms, carried by insects and living not like the round worm, threadworm and hookworm in the intestinal canal—but in the body tissues. These worms are: *Wuchereria bancrofti—Filaria bancrofti*—carried by the ordinary female *culex* mosquito; *Loa loa*, carried by the mangrove fly—*Chrysops*; *Onchocerca volvulus*, carried by the river fly—the black *Simulium* midge.

*Filaria bancrofti* lives in the lymphatic system—mainly in the lymphatic glands. It produces an enormous number of young microfilariae which swarm in the superficial capillaries during the hours of darkness ready to be sucked up by mosquitoes. The glands occupied by the adult filariae are apt to undergo periodic inflammatory changes. Recurring filarial lymphangitis is a common clinical finding in all parts of the tropics. The clinician is generally aided by a history of previous similar attacks. Retroperitoneal lymphangitis may closely simulate an acute surgical emergency and it is most important in dealing with people from the tropics to inquire about similar occurrences in the past and to examine for signs of hydrocele and other evidences of bancroftian filariasis.

The condition may pass on to a stage of elephantiasis, but this is very rare except in natives of the tropics who are subject to nightly assault by hordes of heavily infected mosquitoes and to secondary streptococcal invasion through fissures and sores in the skin.

*Loa loa* infection is not dangerous. The infection is caused by a day-biting fly and the microfilariae are found in the blood in the day-time only. The adult worms live in the subcutaneous tissues, are very active and migratory, and may cross the bulbar conjunctiva from time to time. In former days doctors used to grab the worm with forceps as it crossed the ocular conjunctiva, make an incision and extract the worm. The infection gives rise to skin allergies and to the appearance of transient swellings—Calabar swellings—from a hen's egg to a goose egg in size in various parts of the body—very puzzling to those who are not familiar with the condition. The infection is confined to rain-forest areas of West Africa and is seen not infrequently in visitors from this country.

I must now introduce you to a more serious invader—*Onchocerca volvulus*—conveyed by *Simulium damnosum*—an appropriately named small black midge. In chronically infected persons large cystic easily-shelled-out swellings appear in the subcutaneous tissues, over bony prominences and round joints. They may reach the size of a small coconut.

The cysts contain coiled-up male and female worms. The microfilariae are not found in the blood but in the deeper layers of the skin. In order to establish a diagnosis fine skin shavings are taken and the living microfilariae are seen by the microscope.

The oustanding importance of this worm is the liability to severe eye complications. All the eye tissues are liable to damage. One may get conjunctivitis, keratitis, uveitis and choroido-retinitis often with irreversible changes and blindness. Not without reason has this worm been called *Filaria caecutiens* (the blinding worm) in Guatemala, for in Central Africa and Central America the amount of blindness and visual disability caused by the infection is serious. Europeans are readily attacked. I find that British ladies with their addiction to gardening are singularly careless about applying insect repellents before emerging from their fly-proof houses, thus falling victim to this vicious fly.

It has very recently been realized that the old description of onchocerciasis as " filaria with knobs on " is no longer correct and that the eye may be invaded early by the young of paired but still wandering worms which have not yet settled down in their cystic abodes. Last year I had under my care a European school-child with severe keratitis but with no sign of adult worms. At the present time I have a European lady patient with advanced eye changes, with microfilariae in the anterior chamber and corneae but without any sign of adult worms.

It is therefore incumbent on the practitioner who comes across such eye changes in a recent visitor to or resident in the tropics of Africa and Central America to consider the possibility of onchocerciasis and to refer the patient to an expert for examination by the slit lamp and corneal microscope. Before the eye changes come on, however, there are usaully severe signs of skin irritation or lichenoid eruptions.

*Treatment.* Thanks to American research during the war we have in diethylcarbamazine (hetrazan-banocide) a powerful weapon against *bancrofti* and *loa* infections and a considerable aid in dealing with onchocerciasis. I would however plead for admission of such cases to a tropical-diseases hospital for treatment as a very cautious approach must be made and cortisone must be available for local and general use at the slightest sign of acute reaction in the eye when treatment is begun.

The British Empire Society for the Blind has recently given £40,000 for the relief of onchocerciasis in West Africa.

The use of D.D.T. in small rivers and streams is rapidly controlling the extent of the river-fly pest in certain places. At Jinga in Uganda—hitherto one of the worst areas in the world for this disease—intensive anti river-fly activity has been in operation for some time so that we now feel that it is safe for Her Majesty to go there in the near future to visit the great new dam.

### LEPROSY

I must conclude with some remarks about leprosy. So far as we know no case of leprosy has developed in this country for many years though there are numerous imported cases. I am not sure that such a happy state of affairs is likely to persist. Until recently leprosy was not a notifiable disease. It was realized that changed political conditions in India would lead to the emigration of Eurasians or Anglo-Indians in considerable numbers and that some of these would have leprosy. There are about 200 known cases in this country—how many there are unrecognized we cannot tell.

Now leprosy cases are notified in strict secrecy to the Ministry of Health in London, and after a visit from an expert the local Medical Officer of Health is informed verbally and strictly confidentially by him. But he can do nothing about it. The waiter may go on waiting, the school-teacher may teach infants, and the mother may go on producing children and probably infecting them without let or hindrance. The Minister of Health has no reserve powers of compulsory segregation of infective cases. It is true that one leprosy hospital exists at Reigate, Surrey—a branch of the London Hospital for Tropical Diseases with accommodation for twenty cases—shortly to be expanded to twenty-six. There is also St. Giles' home in Essex cared for by a band of nuns. But no one can be compelled to go there.

A high porportion of leprosy patients in this country are infective but it must be realized that for the spread of the disease to occur prolonged and intimate contact—as from mother to child—is usually necessary, and that most patients acquire their disease in infancy—but not always. We have three European patients at present who did not visit the tropics till adult life.

In persons returning from the tropics the diagnosis of leprosy should always be kept in mind in connection with complaints of numbness, tingling or unusual rashes and above all in areas of depigmentation with anaesthesia. In no circumstances should the word leprosy be mentioned to the patient for the social implications are so severe. The patient should be referred to an expert (the Ministry will always advise) without his knowing the nature of the suspected illness. A skin biopsy will generally be required to determine the diagnosis—and prognosis. The patient should be completely stripped. We have at the present time a European Military Officer, a British soldier, an Anglo-Indian typist and a Maltese school-teacher who look completely normal in every way when fully dressed; their lesions are all hidden by their clothing.

Despite what you read in newspaper propaganda, leprosy is a very difficult and indeed dangerous disease to treat in Europeans, Anglo-Indians or other half-castes. Modern sulphone therapy is beset with difficulties; it is quite easy to produce a severe or even fatal reaction in the nerves with quite a small dose of sulphone preparation with high fever, acute strangulation of the nerve fibres with complete paralysis and dangerous eye changes.

Many contretemps are met in the course of treatment which at best is of several years' duration—great patience is needed by both patient and doctor.

### CLOSING REMARKS

I have no time to deal with the short fevers, such as dengue and sandfly fevers which may now appear here with fast air transport—the vectors are not found here and they will not spread. The possibility of importing bubonic plague by a passenger from Bombay is well recognized and is one reason for surveillance of an M.O.H. of any illness occurring within a fortnight of arrival in this country.

The British Commonwealth and Empire is composed of an aggregate of free and equal peoples but, as in *Animal Farm*, some are more equal than others. The most important inequality which concerns us is that alone among the members of the Commonwealth Great Britain has no powers of exclusion. The introduction of such powers to enable us to shut out criminal and infected persons from other parts of the Commonwealth would, as the Home Secretary said the other day, offend against the sacred principle of the door open for all Her Majesty's subjects. We must therefore continue to expect an inflow of leprosy and other tropical diseases and to be prepared to diagnose and treat them.

